# Molecular Signatures of Dissolved Organic Matter Generated from the Photodissolution of Microplastics in Sunlit Seawater

**DOI:** 10.1021/acs.est.1c03592

**Published:** 2023-11-13

**Authors:** Aron Stubbins, Lixin Zhu, Shiye Zhao, Robert G. M. Spencer, David C. Podgorski

**Affiliations:** † Department of Marine and Environmental Sciences, Northeastern University, Hurtig 102, Boston, Massachusetts 02115, United States; ‡ Departments of Civil and Environmental Engineering, and Chemistry and Chemical Biology, Northeastern University, Hurtig 102, Boston, Massachusetts 02115, United States; § Research Institute for Global Change, Japan Agency for Marine-Earth Science and Technology (JAMSTEC), Yokosuka 237-0061, Japan; ∥ Department of Earth, Ocean and Atmospheric Science, Florida State University, Tallahassee, Florida 32306, United States; ⊥ Pontchartrain Institute for Environmental Sciences, Department of Chemistry, Chemical Analysis & Mass Spectrometry Facility, University of New Orleans, New Orleans, Louisiana 70148, United States

**Keywords:** microplastics, photochemistry, dissolved organic matter, polystyrene, polypropylene, polyethylene, FT-ICR MS

## Abstract

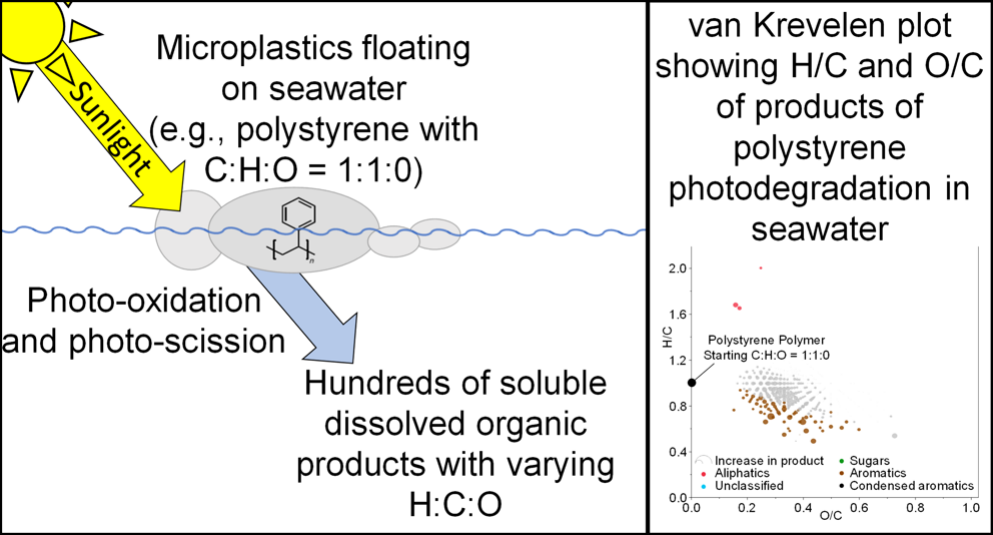

Plastics are accumulating on Earth, including at sea. The photodegradation of microplastics floating in seawater produces dissolved organic matter (DOM), indicating that sunlight can photodissolve microplastics at the sea surface. To characterize the chemistry of DOM produced as microplastics photodissolve, three microplastics that occur in surface waters, polyethylene (PE), polypropylene (PP), and expanded polystyrene (EPS), were incubated floating on seawater in both the light and the dark. We present the molecular signatures of the DOM produced during these incubations, as determined via ultrahigh-resolution mass spectrometry. Zero to 12 products were identified in the dark, whereas 319–705 photoproducts were identified in the light. Photoproduced DOM included oxygen atoms, indicating that soluble, oxygen-containing organics were formed as plastics photodegrade. PP and PE plastics have hydrogen-to-carbon (H/C) ratios of 2 and generated DOM with average H/C values of 1.7 ± 0.1 to 1.8 ± 0.1, whereas EPS, which has an H/C of 1, generated DOM with an average H/C of 0.9 ± 0.2, indicating the stoichiometry of photoproduced DOM was related to the stoichiometry of the photodegrading polymer. The photodissolution of plastics produced hundreds of photoproducts with varying elemental stoichiometries, indicating that a single abiotic process (photochemistry) can generate hundreds of different chemicals from stoichiometrically monotonous polymers.

## Abstract 2

We present a chemical description of the hundreds of byproducts formed when sunlight breaks down plastics in seawater.

## 1 Introduction

Industrial plastic production began in the 1950s and has accelerated ever since.^1^ The durability and increasing production of plastics, coupled with a lack of large-scale reclamation strategies, has allowed plastics to accumulate to become a significant, anthropogenic, carbon-based material in the Earth system.^2^ Different plastic polymers have different carbon contents. Polyethylene (PE) and polypropylene (PP) are 86% carbon by mass based upon their elemental stoichiometry, while polystyrene (PS) is 92% carbon by mass.^2^ These three polymers together accounted for 72% of plastic carbon production between 2002 and 2014^1^ and presumably dominate the pool of plastic carbon on Earth.

In the ocean, 15 to 51 trillion plastic items, dominated by PE and PP microplastics, have accumulated at the sea surface.^3,4^ Despite this accumulation, only ∼1% of estimated annual inputs to the oceans are found afloat at sea.^3−5^ An estimated 40% of plastics entering the oceans should sink as they are formed from polymers with densities greater than that of seawater.^6^ The other 60% of plastic entering the oceans is made from buoyant polymers. Buoyant plastics include PP and PE, which have intrinsic densities lower than seawater, plus foamed plastics such as expanded PS (EPS).^7^ Removal mechanisms for buoyant macro and microplastics have been proposed, including consumption by marine life,^8^ biofouling and/or aggregation with organic detritus leading to sinking,^9,10^ deposition in under-sampled remote locations,^11^ under-sampling of megaplastics,^12^ and degradation to small particles, solutes and gases^13−16^ that are not captured by the tow nets and in situ pumps generally used to sample plastics at sea (i.e., these nets and pumps only capture particles and most often only down to a size of approximately 300 μm).^3,4,13−16^

Away from the ocean, the degradation of plastics has been studied for decades. Processes include bio- (mainly microbial), thermo-, and photodegradation.^17^ Bio- and thermo-degradation are slow compared to sunlight-driven photodegradation under ocean conditions, making exposure to sunlight the most important factor in determining the rate of plastic degradation in surface waters.^18^ Photodegradation reduces polymer molecular weight through scission reactions,^18^ forms novel nonoligomer structures through cross-linking reactions (i.e., the formation of covalent bonds between polymer carbon atoms),^19^ oxidizes the polymer hydrocarbons, and produces gaseous products such as methane, ethylene,^15^ carbon monoxide (CO), carbon dioxide (CO_2_), and a suite of low-molecular-weight and oxidized products,^18,20^ some of which are soluble and some of which can be utilized by microbes.^17,21^

In the ocean, as plastics photodegrade, the polymer also dissolves (i.e., photodissolves), producing dissolved organic matter (DOM).^13^ Ocean DOM is a major pool of global carbon similar in quantity to the atmospheric CO_2_ pool^22^ and is an important source of organic carbon to marine microbes.^23^ The DOM released from plastics as they photochemically dissolve (photodissolve) in seawater can both stimulate and inhibit microbial growth,^13,16^ suggesting that the photodissolution of plastics could impact carbon cycling and microbial ecology in ocean surface waters.

Although the photodissolution of plastics to DOM is recognized, the chemical quality of the DOM produced as plastics dissolve is poorly understood. Here, we present data concerning the chemistry of soluble organics (i.e., DOM) produced as plastics photodissolve in seawater. This information is of importance to environmental scientists and engineers who wish to understand what happens to plastics in the environment, what soluble byproducts plastics release, and the fate and impact of those soluble byproducts. Specifically, we utilized ultrahigh-resolution Fourier transform ion cyclotron mass spectrometry (FT-ICR MS) to provide molecular formula level information about the DOM produced during the dark and light incubations of three buoyant polymers commonly found in the surface ocean: PE, PP, and EPS.^6,13,24^ We hypothesized that (1) few to no formulas would be produced in the dark based upon the insolubility of these polymers in water;^25−27^ (2) all plastics would yield photoproducts as they photodissolved; (3) that photoproduced molecular formulas would include oxygen, indicating that photo-oxidation is important to the photodissolution of the plastics, and (4) that the polyolefins, PE and PP, which have atomic hydrogen-to-carbon ratios (H/C) of 2 would generate higher H/C DOM than the aromatic polymer, EPS, which has an H/C of 1.^2^

## 2 Experimental Section

### 2.1 Microplastic Preparation and the Photochemical Experiment

The details of the microplastic samples, their preparation, and their irradiation are presented elsewhere.^13^ In brief, postconsumer PP (NIVEA facial cleanser bottle) and EPS (disposable lunch box)^13^ were cut into small pieces (average size: 3.0 ± 0.9 mm). For PE, a standard PE granule was purchased (nominal diameter: 2 mm; PN: ET306300/1, Goodfellow). All laboratory plasticware was cleaned by triple rinsing with ultrapure water (Milli-Q), soaking overnight in ∼pH 2 water (4:1000, v:v, 6 N HCl:Milli-Q), triple rinsing with Milli-Q, and then drying. Glassware and quartzware were cleaned as above and then ashed at 450 °C for 6 h to remove trace organics. Seawater (salinity ∼35) was collected from an ∼5 m depth in the South Atlantic Bight using Niskin bottles aboard the RV *Savannah* and gravity-filtered (0.2 μm; AcroPak 1500, PALL) directly into precleaned 20 L high-density PE carboys. To remove natural, photochemically active organics before adding microplastics, seawater was transferred to 2 L ashed quartz flasks and placed under germicidal ultraviolet-C light until the DOM concentration was stable.

Microplastics were cleaned by sonification in Milli-Q water to simulate prior exposure to water, as expected for plastics found at sea. Microplastics were analyzed previously via elemental analysis to determine their percent carbon by mass^13^ (Table 1). The theoretical percent carbon by mass was also calculated from each polymer’s elemental formula (Table 1). Comparison of measured and theoretical percent carbon by mass for polymers provides some indication of purity. However, for the measured value to deviate from the theoretical, impurities, including additives, need to have different carbon:mass ratios compared to the polymer and to be present at high enough concentrations for any difference in the carbon:mass ratio between the additive and the polymer to result in a detectable difference in the measured percent carbon by mass of the plastic. The latter detectable difference is impacted by analytical errors from gravimetric analysis of mass and elemental analysis of carbon. Within these limitations, the measured percent carbon by mass for PE and PP was within error of their theoretical values, providing no definitive evidence of mass or carbon contributions from additives. The measured value for EPS was 2.0 ± 1.0% lower than the theoretical value for pure polystyrene (Table 1), suggesting additives could have contributed additional mass to the polymer.

**Table 1:**
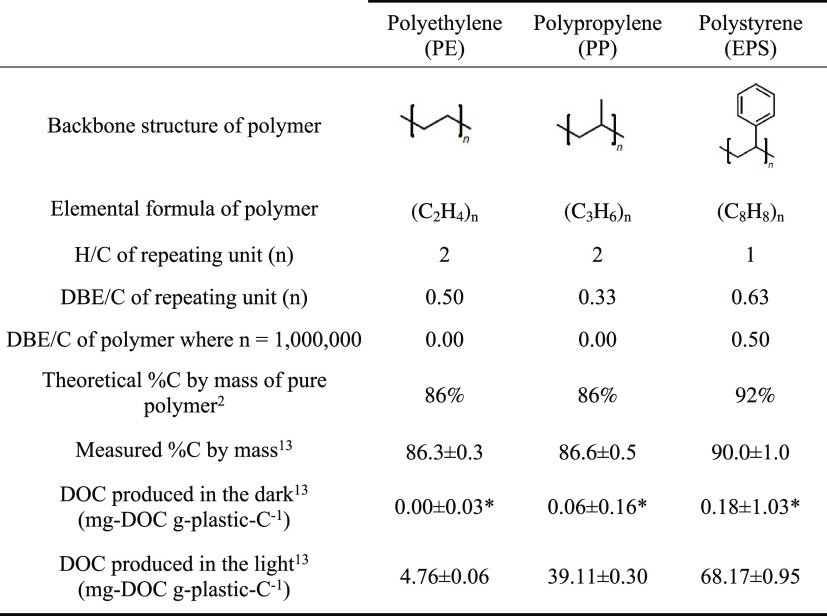
Summary of Polymers Used, Including Their Backbone Structures, Elemental Formulas, Stoichiometries, and Theoretical and Measured Percent Carbon (C) by Mass, Plus the Amount of Dissolved Organic Carbon (DOC) Produced during 54-Day Dark and Light Incubations

Note: ± standard deviation.

* Indicates value was not significantly different from zero using a *t* test. DBE = double bond equivalents.

Four hundred eighty cleaned pieces of each polymer were randomly selected, weighed (XP26 DeltaRange, Mettler Toledo), and divided into two groups (240 particles per group). This yielded a total of six microplastic aliquots: 2 × PE, 2 × EPS, and 2 × PP. These aliquots were rinsed three times with Milli-Q, three times with the previously photobleached and sterile-filtered seawater, and then transferred into the 2 L ashed and ultraviolet-C-sterilized spherical quartz irradiation flasks with 1 L of photobleached seawater (two flasks for each plastic type = 6 flasks). Two control flasks were filled with photobleached seawater without plastics, resulting in a total of 8 flasks. Half of the flasks (i.e., one of each treatment) were wrapped in heavy-duty aluminum foil to provide dark controls. All flasks were then placed inside a solar simulator.

Irradiations were conducted in a solar simulator with 12 UVA-340 bulbs (Q-Panel), which provides light with a spectral shape and flux approximating natural solar irradiance from 295 to 365 nm.^28^ This wavelength range is responsible for most environmental photochemical reactions, including plastic photodegradation.^29−31^ The integrated irradiance (14 ± 0.7 W m^–2^) in the solar simulator was quantified using a spectroradiometer (OL 756, Optronic Laboratories) calibrated with a National Institute of Standards and Technology (NIST) standard lamp (OL752-10 irradiance standard).^32^ One day of irradiation under the solar simulator equaled ∼1.27 times the daily solar irradiance received by the subtropical ocean gyre surface waters^33^ where microplastics accumulate,^4^ and about 0.67 times the daily solar irradiance at the equator.^34,35^ A side-mounted fan maintained temperatures between 25 and 30 °C. These temperatures are similar to surface seawater temperatures in the subtropical gyres where floating microplastics accumulate.^4^ For instance, sea surface temperatures range from approximately 21 to 27 °C in the North Atlantic^36^ and the North Pacific^37^ subtropical gyres. The flasks were repositioned daily to average the potential spatial variation in the light flux under the solar simulator. As previously reported, flow cytometry for dark and light samples confirmed the samples remained sterile, giving confidence that observed changes in DOM were due to abiotic processes.^13^

### 2.2 Dissolved Organic Carbon Production for FT-ICR MS Analyses

**Figure 1:**
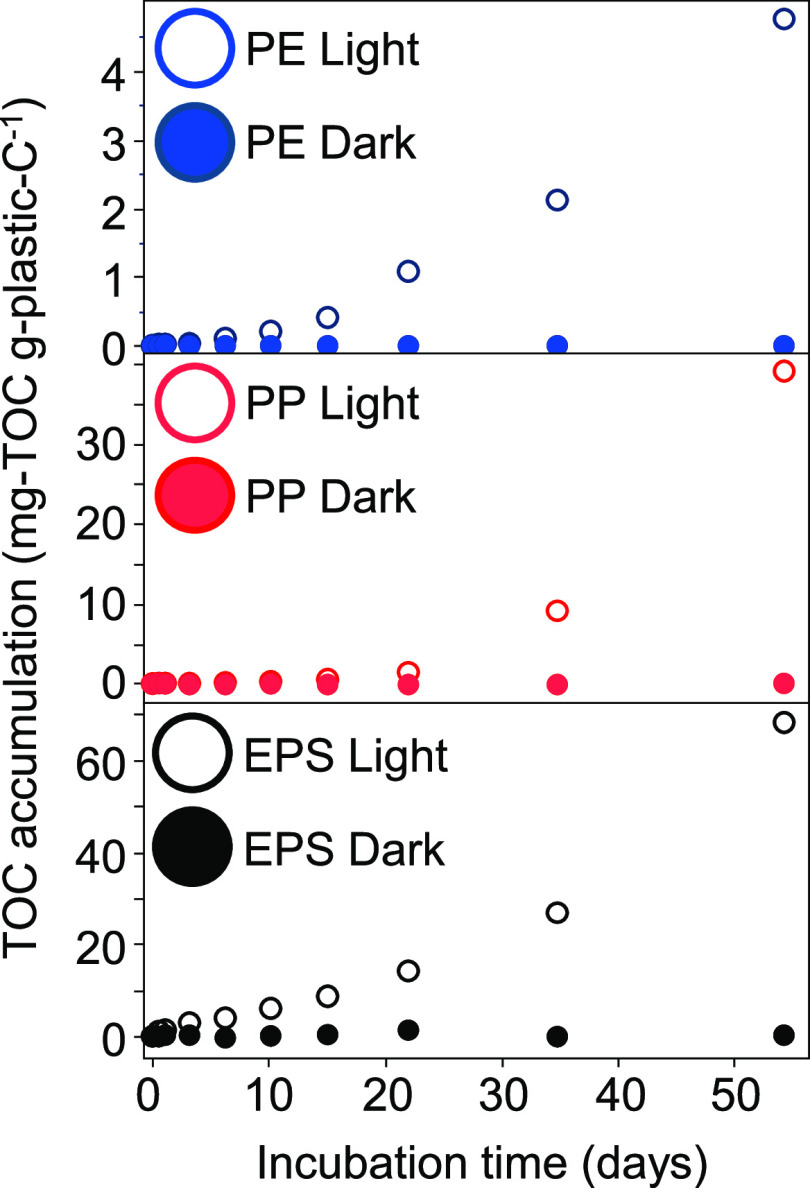
Total dissolved organic (TOC) production from polyethylene (PE), polypropylene (PP), and expanded polystyrene (EPS) microplastics floating on seawater in both light and the dark.

DOM concentrations were determined as both total and dissolved organic carbon (TOC and DOC). For TOC samples, analyzed throughout the incubations to generate time series data, flasks were gently mixed to homogenize water, then ∼20 mL of water was collected using precombusted Pasteur pipettes and acidified to pH < 2 using HCl (HPLC grade) before analysis using a Shimadzu TOC-VCPH analyzer.^38^ Time series data for TOC (Figure 1) have been published previously.^13^ Certified DOC standards (low carbon seawater, LSW, and deep seawater reference material, DSR) from the Consensus Reference Materials (CRM, University of Miami) were measured to confirm precision and accuracy. Measured DSR values were consistent with the consensus value (0.49–0.53 mg L^–1^) with a standard deviation <5%. Routine DOC detection limits are 0.034 ± 0.0036 mg L^–1^, and standard errors are typically 1.7 ± 0.5% of the DOC concentration.^38^

After 54 days, TOC levels in all light treatments had increased sufficiently to suggest that photoproducts would be detectable via FT-ICR MS. Thus, the experiments were finished and samples were collected and filtered through precleaned GHP 0.2 μm syringe filters (PALL), acidified to pH < 2, and analyzed for DOC using the Shimadzu TOC-VCPH analyzer. All 54-day dark and light samples were thus analyzed for both TOC (unfiltered) and DOC (filtered), with no differences between these two methods (paired *t* test: *p* > 0.05, *n* = 12). The filtered samples were used for FT-ICR MS analyses and to calculate DOC production after 54 days (Table 1), the latter being calculated as DOC values after 54 days minus time zero DOC. All DOC analyses were run on duplicate samples taken from a single flask. Errors accompanying DOC production in Table 1 are standard deviations calculated from the errors from duplicate samples collected at both time zero and at 54 days. The concentrations of TOC and DOC in the light plastic-free controls, the dark plastic-free controls, and in each dark sample with plastics added remained constant, whereas exposure of all plastics to light produced TOC (Figure 1) and DOC (Table 1).^13^ After 54 days of irradiation, EPS produced the most DOC per mass of irradiated plastics, followed by PP and PE (Table 1). The results for TOC accumulation in these experiments are published elsewhere^13^ and are repeated here to illustrate the experimental design used to generate DOM for FT-ICR MS analyses. A full discussion of the TOC data, including kinetics of plastics photodissolution was published previously.^13^ The DOM samples collected after 54 days were processed for FT-ICR MS analyses as described below.

### 2.3 Fourier Transform Ion Cyclotron Resonance Mass Spectrometry (FT-ICR MS)

The sample volumes remaining at the end of irradiations and after DOC analysis (293–433 mL) were syringe-filtered through 0.2 μm Acrodisc GHP filters, acidified to pH 2 using HCl (HPLC grade), and solid-phase-extracted using PPL.^39^ Extraction efficiencies ranged from 25 to 53% (Section 3.1). Aliquots of solid-phase extracts were diluted in methanol to 60 μg of C mL^–1^ and infused into an electrospray ion source at 700 nL min^–1^. Negatively charged ions were analyzed with an FT-ICR mass spectrometer equipped with a 21 T superconducting magnet housed at the National High Magnetic Field Laboratory, Tallahassee, Florida.^40^ 100 broadband scans were accumulated per spectrum. Mass spectra were internally calibrated using naturally present compounds in DOM as calibrants.^41,42^ Molecular formulas were assigned to detected masses based on published rules.^43,44^ For each molecular formula, we calculated the aromaticity index (AI)^45,46^ as
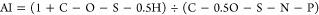
1values 0.5 to 0.67 and >0.67 were assigned as aromatic and condensed aromatic structures, respectively.^45^ Further compound classes were defined as follows: Unclassified = AI < 0.5, H/C < 1.5, O/C < 0.9; Aliphatics = H/C 1.5 to 2.0, O/C < 0.9, N = 0; and; Peptide molecular formulas = H/C 1.5 to 2.0, O/C < 0.9 and *N* > 0; and Sugars, O/C > 0.9.^34,47^ It should be noted that compounds identified as “peptides” have the molecular formulas of peptides, and “sugars” have the molecular formulas of carbohydrates, but isomers represented by these formulas and others can have a variety of structures. Further, each of these chemical categories should be regarded as a guide to the potential, rather than definitive, chemical structures represented by the formulas.^34^ Carbon normalized double bond equivalents (DBE/C) were calculated as
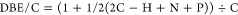
2Relative abundance was calculated as the intensity (i.e., peak intensity as recorded by FT-ICR MS) of an individual formula’s peak divided by the average intensity of all assigned formulas within a sample. This results in the average intensity of formulas in each sample being equal to 1. A full list of all formulas assigned, including their relative abundance in each sample is presented in Table S1.

Despite a lack of DOC production in the dark (Table 1),^13^ we sought to identify any dark products by comparing FT-ICR MS data for the dark incubations of the plastics with dark seawater controls. To identify photoproduced formulas, we treated the dark incubations of plastics and the irradiation of plastic-free seawater as dual controls. A van Krevelen plot for the plastic-free seawater is provided (Figure S1), and data for all light and dark samples, including controls, are provided in the SI (Table S1). To reduce the possibility of identifying false positives, strict criteria were applied to define both light and dark products. Specifically, for a formula to be defined as a product, it had to increase in relative abundance by at least 2 compared to the controls (i.e., relative abundance of a product had to increase by at least 2 times the mean abundance of all formulas in a sample) and must also quadruple in abundance relative to the same formula’s abundance in the relevant control samples. These two criteria are distinct and serve to rule out two types of false positives. The stipulation that relative abundance must increase by at least 2 ensures that small fluctuations in abundance for formulas with initial abundances close to the detection limit do not lead to false positives as could occur if only the percentage increase criteria were applied. The stipulation that abundance must increase by 300% acts to reduce the likelihood of identifying false positives for formulas with high initial abundance for which a change in abundance intensity of 2 would be a minor increase compared to its initial abundance. This conservative approach identified 0 (zero)-12 products in the dark and 319 to 705 products in the light (Table 2, Table S1).

**Table 2:** Summary of the Molecular Quality of Dissolved Organic Matter Produced during the Dark and Light Incubation of Polyethylene (PE), Polypropylene (PP), and Expanded Polystyrene (EPS)

	light			dark		
	PE	PP	EPS	PE	PP	EPS
total formulas	319	705	406		12	
CHO formulas	279 (87.5%)	700 (99.3%)	406 (100%)		12 (100%)	
CHON formulas	11 (3.5%)	5 (0.7%)				
CHOS formulas	29 (9.1%)					
aliphatic formulas	307 (96.2%)	698 (99%)	3 (0.7%)		11 (91.7%)	
unclassified formulas	12 (3.8%)	2 (0.3%)	339 (83.5%)		1 (8.3%)	
sugars formulas						
aromatics formulas			63 (15.5%)			
condensed aromatics formulas			1 (0.3%)			
peptide-like formulas		5 (0.7%)				
average molecular mass (Da)	372 ± 99	458 ± 130	372 ± 96		292 ± 20	
average H/C	1.8 ± 0.1	1.7 ± 0.1	0.9 ± 0.2		1.8 ± 0.1	
average O/C	0.39 ± 0.11	0.34 ± 0.1	0.38 ± 0.11		0.24 ± 0.02	
average DBE/C	0.17 ± 0.08	0.17 ± 0.06	0.59 ± 0.08		0.18 ± 0.07	

Note: ± standard deviation. % indicates the percentage of product formulas that fall within the respective class. – indicates no products in this class were detected. DBE = double bond equivalents.

## 3 Results and Discussion

### 3.1 DOC Production, Extraction Efficiency, and Analytical Windows

As noted in the methods and discussed elsewhere,^13^ the concentrations of DOC in the dark incubations remained constant, whereas exposure of all plastics to light produced DOC (Table 1).^13^ Other studies have noted leaching of DOC in the dark when plastics first contact seawater.^16^ In the current work, plastics were precleaned to assess whether plastics themselves dissolve in seawater rather than to assess whether plastics can leach sorbed compounds. The lack of quantifiable DOC accumulation in the dark for our experiments indicates that plastics either do not dissolve or dissolve very slowly in the dark. This result is in keeping with decades of research that has shown PE, PP, and EPS to be insoluble in water.^25−27^ Blending of C–C bonded polymers such as PE, PP, and EPS with both starch and pro-oxidants produces novel, blended polymers susceptible to oxidation and presumably dissolution at moderate temperatures,^17^ but these modified polymer forms were not the focus of the current study.

After 54 days of irradiation in the light, EPS produced the most DOC per mass of plastics, followed by PP and PE (Table 1). The higher photodissolution rate for EPS is to be expected given polystyrene is an aromatic polymer capable of absorbing ultraviolet sunlight, while PP and PE are alkanes that lack moieties within the pure polymer that can absorb ultraviolet sunlight.^2,18^ The initial photoreactivity of PP and PE has been proposed to stem from the presence of impurities sorbed to the polymer, such as additives, and/or the presence of impurities in the polymer resulting from the oxidation of the polymer during processing (e.g., heating may cause oxidation during extrusion or molding).^2,18^ For photoreactions of natural DOM, aromatics are also the main chromophores and are preferentially photodegraded compared to bulk DOC and when compared to aliphatic components of the DOM pool.^31,44^

The efficiency of DOC recovery via solid-phase extraction using PPL cartridges was 38% for the dark and light plastic-free controls. DOC recoveries for dark samples with plastics added ranged from 39 to 48%, while DOC recoveries for light samples with plastics added showed the greatest variability with recoveries of 53%, 43%, and 25% for EPS, PP, and PE, respectively. These latter data suggest the chemistries of DOM from photodissolved plastics may have varied sufficiently to have impacted their PPL recoveries. However, as we could not analyze the nonextractable fraction, it is not discussed further. Future studies may seek to define other fractions of the total DOM pool released from plastics by using other isolation techniques such as reverse osmosis coupled to electrodialysis.^48^ However, no available method can isolate 100% of the DOM pool from the salt background of seawater and a fraction of DOM always goes unseen.^49^ This limitation is true for any analytical method. All of the methods, including the methods applied here, have a defined analytical window. Critically, the same analytical window was applied to all samples in a way that was consistent with other research in the field. For DOC, our analytical window is defined as organic carbon that passes through a 0.2 μm filter and is then oxidizable to carbon dioxide via high-temperature chemical oxidation. For FT-ICR MS data, our window provides a view of the PPL extractable component of the DOM pool that is ionizable via electron spray ionization in negative-ion mode and detectable under the specific conditions applied within the FT-ICR mass spectrometer. For both DOC and FT-ICR MS data, these analytical windows are widely applied in environmental science and provide data that is valuable for direct comparison between samples both within and across studies, providing data that has advanced our understanding of the source, nature, and reactivity of DOM in the aquatic environment.^22,50,51^

### 3.2 Molecular Formulas of Dark Products

Twelve products were identified in the dark incubations of PP, and no products were identified in the dark incubations of PE and EPS (Table 2). The identification of zero to 12 product formulas in the dark compared with the hundreds of product formulas identified in the light (Table 2) is consistent with the lack of DOC accumulation in the dark (Table 1)^13^ and indicates these plastics are stable at both the bulk carbon and molecular level in the dark in agreement with the definition of these polymers as insoluble in water.^25−27^ The 12 formulas assigned as dark products for PP incubations were all CHO formulas with low mean molecular mass (292 ± 20 Da), high H/C (1.8 ± 0.1), and low O/C (0.24 ± 0.02; Table 2). These few formulas could represent analytical noise, products formed in the dark, or impurities or additives that were not completely cleaned from plastics during ∼24 h soaking during cleaning but were leached during the longer (54-day) dark incubations.

### 3.3 Molecular Formulas of Photoproducts

**Figure 2:**
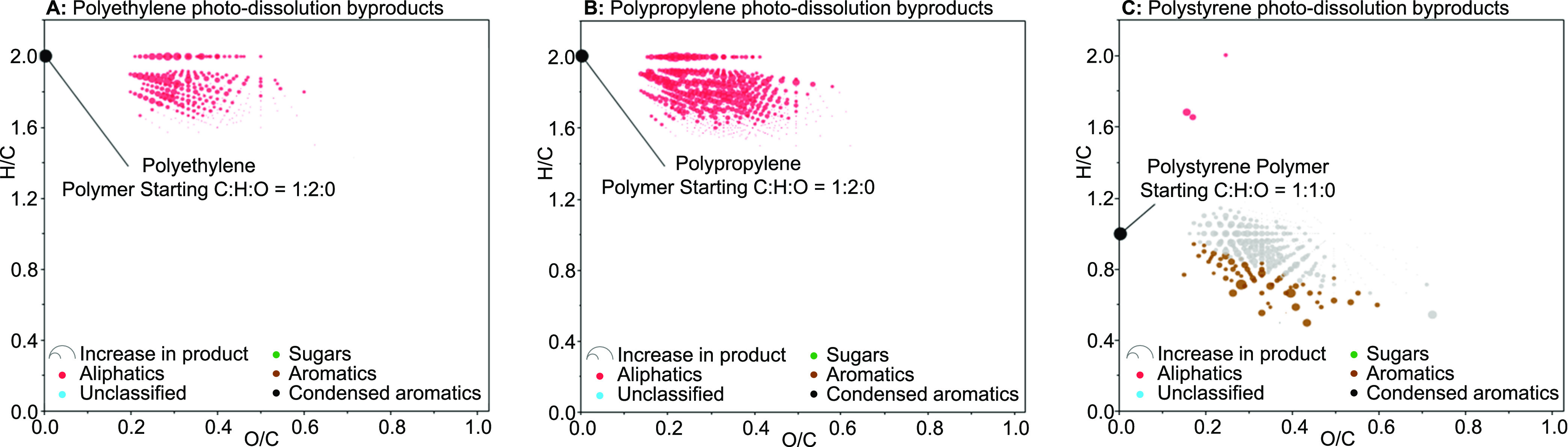
van Krevelen diagrams for CHO-only molecular formulas produced during the photodissolution of the following polymers in seawater: (A) polyethylene; (B) polypropylene; and (C) polystyrene. Color indicates the compound classes assigned to formulas, and the size of the markers indicates the relative production of the formula (i.e., a bigger marker indicates greater production). Marker in black on the *y*-axis (i.e., O/C = 0) indicates the C/H/O of the polymers.

Hundreds of photoproducts formed during the irradiation of each polymer (Table 2; Figure 2). A small number of photoproduced formulas contained N and S, indicating that N and S may be incorporated into DOM as plastics photodegrade but that the majority of DOM produced contains only C, H, and O atoms (Table 2). N and S in photoproducts could derive from natural organics in the seawater, from the photochemical incorporation of inorganic N and S from the seawater (e.g., from nitrate and sulfate), or from additives or contaminants bound to the plastics being photosolubilized. The N- and S-containing molecular formulas produced from plastics were few in number and generally similar in CHO stoichiometry to the CHO-only formulas (Figure S1). Thus, we combine all of the formulas together in the following discussions.

All formulas included oxygen heteroatoms (Figure 2) and the average molecular masses of photoproducts were 372 Da for PE and EPS and 458 Da for PP (Table 2) as expected for organics in our analytical window (i.e., water-soluble organics that are extractable via PPL and ionizable via negative-ion electrospray ionization). These results indicate that photodegradation while floating on seawater led to the oxidation and scission of the high-molecular-weight, insoluble, hydrocarbon polymers to yield soluble, lower-molecular-weight, oxidized products.

The standard deviation in average molecular weight and average O/C of photoproducts was greater than the differences between samples (Table 2). The main difference between samples was in the average H/C and associated metrics, including DBE/C and AI (Table 2). Specifically, the EPS photoproducts had lower average H/C and higher average DBE/C compared to those of PE and PP photoproducts (Table 2). These trends follow those of the original polymers, with the aromatic polymer EPS having an H/C of 1 and higher DBE/C values, while the polyolefins PP and PE both have an H/C of 2 and lower DBE/C values (Tables 1 and 2; Figure 2). For the polyolefins, the upper bound to the H/C of the observed photoproducts is approximately 2, which coincides with the H/C of the polymer. There are no rings to cleave or Cs to saturate in the polyolefins’ alkane structures. Thus, H cannot be added to increase the H/C without adding other elements making it unlikely that organic molecules with H/C values >2 will form during oxidation of PE and PP. For EPS, with a starting H/C of 1 due to its aromatic backbone, ring cleavage can increase H/C (increase saturation), while oxidation, cross-linking, and scission reactions can all decrease H/C. Photoproducts formed from EPS did indeed range from lower to greater than 1 (Figure 2). The lower average H/C for EPS photoproducts (0.9 ± 0.2; Table 2) compared to the starting polymer suggests that oxidation, cross-linking, and/or scission reactions may have been more important in EPS photodissolution than ring cleavage reactions. Thus, the H/C trends in the average molecular formula properties of the DOM produced during the irradiation of EPS, PE, and PP were consistent with the scission and photo-oxidation of each polymer.

Molecular formulas were classified by compound class based upon elemental stoichiometries.^34,47^ Resultant trends were reflective of those of the original polymers. Over 99% of formulas photoproduced from the aliphatic, higher H/C polyolefins, PP and PE, were assigned as either aliphatic or unclassified compounds (Table 2; Figure 2), while the photoproducts of the aromatic EPS yielded DOM enriched in lower H/C compounds classes, including aromatic (15.5%) and condensed aromatic (0.25%) formulas. Aromatic moieties within natural DOM, including those identified via FT-ICR MS,^44,47^ are usually preferentially photodegraded by sunlight.^31^ Thus, the photoproduction of aromatic DOM from EPS suggests that the photoproduction of aromatic DOM exceeded any subsequent photodegradation of these dissolved aromatic compounds. Further work should assess whether photoresistant aromatic compounds are formed as EPS photodissolves or whether once a source of these aromatics disappears (i.e., all EPS is removed), these dissolved aromatic products are further photodegraded to nonaromatic DOM or carbon dioxide.

Previous studies have shown the DOC photoproduced from PE, PP, and EPS to be relatively biolabile.^13,16,21^ The photoproduced DOM samples analyzed here via FT-ICR MS were previously submitted to biodegradation experiments, and the results have been published.^13^ In summary, DOM from the EPS (76 ± 8% biolabile DOC) and PP (59 ± 8% biolabile DOC) samples was as biolabile as some of the most biolabile forms of DOM from natural sources, such as phytoplankton cultures (40–75% biolabile)^52^ and permafrost thaw waters (∼50% biolabile),^53^ indicating that photodegradation of these EPS and PP microplastics yielded DOM that could be rapidly utilized by microbes at the sea surface. High H/C, aliphatic formulas in natural DOM are often among the most biolabile signatures, while aromatic formulas are often less biolabile.^53^ Thus, the enrichment of DOM from PP in high H/C formulas is consistent with the high bioavailability of its DOC, while the high bioavailability of EPS-derived DOC is somewhat at odds with the high aromatic content of this sample. Although aromatic formulas can be more resistant to biodegradation than other formulas in natural DOM samples,^53^ this trend is not universal and can also vary with the type of aromatics studied. For instance, lignin-derived phenols and some aromatic formulas in FT-ICR MS data for Amazon River samples were both biolabile,^54^ while simpler, lower-molecular-weight aromatics can also be readily used as substrates by bacteria.^55,56^

The PE-derived DOM analyzed here via FT-ICR MS is for the PE standard noted in Zhu et al.^13^ The DOM photoproduced from this PE standard was less biolabile (22 ± 4% biolabile DOC) than the DOM from PP and EPS, and also inhibited microbial growth.^13^ However, the chemical signature of this DOM (Figure 2) is similar to that of the biolabile PP-derived DOM and has a similar distribution in van Krevelen space as biolabile DOM from other environments.^53^ Thus, the FT-ICR MS data do not help elucidate why the photoproducts formed from this particular sample of PE inhibited microbial growth. Further studies are required to determine why the photoproducts of some plastics, including some plastic samples of the same polymer type, inhibit microbial growth while others stimulate it. The chemicals inhibiting microbial activity may have derived from additives in this specific PE sample or from contaminants picked up by this sample during manufacture, packaging, or transfer. Identifying the exact molecules or classes of compounds involved in microbial inhibition will likely involve advanced screening methods, such as untargeted analysis of known toxicants^57^ and effect-directed analysis^58^ to identify unknown toxic compounds. For instance, the latter was recently applied to identify the previously undocumented oxidation products of a tire additive to a toxic form that causes paralysis in coho salmon.^59^ Elucidating currently unknown toxicants within the array of molecular products formed as plastics photodegrades is critical to understanding and mitigating the impact of plastics and their degradation products on the ecosystem and human health.

Photodegradation of the polymers produced hundreds of molecular formulas that spanned a range of C:H:O stoichiometries (Table 2; Figure 2). Products were of lower molecular mass than their parent polymers and their formulas included oxygen. These results indicate that the exposure of low-chemical-diversity polymeric hydrocarbons (i.e., plastics) to sunlight in seawater resulted in the production of a suite of chemically diverse, oxygen-containing, soluble photoproducts. A previous study exposed HDPE film to sunlight in air, extracted water-soluble products, and analyzed these products via ESI-Orbitrap MS noting the production of a diverse suite of high H/C (i.e., H/C between 1 and 2) products with an average mass of 460 Da.^21^ Despite differences in experimental and analytical design, these results are similar to those of the PE photoproducts reported here (Table 2; Figure 2). To our knowledge, ultrahigh-resolution data for the soluble photoproducts of EPS and PP have not been previously published.

The diversity of low-molecular-weight, oxidized, soluble photoproducts generated when structurally uniform, high-molecular-weight, insoluble hydrocarbon polymers photodegrade results from several processes. EPS absorbs ultraviolet light at environmentally relevant wavelengths (i.e., wavelengths ≥280 nm).^60^ However, pure PE and PP do not absorb ultraviolet light above 280 nm, and therefore should not undergo direct photo-oxidation at the Earth’s surface.^17^ However, thermal oxidation of polymers during manufacturing and processing produces low levels of carbonyl, hydroperoxide, and other O-containing groups. Carbonyls can absorb sunlight and transfer the energy absorbed to hydroperoxides that drive further photo-oxidation.^17,61^ In addition, the presence of additives and contaminants, such as PAHs, sorbed to plastics may also allow for the initiation of photoreactions.^20,62,63^ Absorbance of ultraviolet light leads to radical formation and the incorporation of oxygen to form carbonyl groups.^64^ Further irradiation leads to Norrish type I or II degradation and the cleavage of C–C bonds, yielding low-molecular-weight, oxidized products of higher solubility^64^ that are likely to occur in our analytical window.

Our results for PE and PP indicate that these polyolefins yielded oxidized aliphatics formulas and unclassified formulas (Table 2; Figure 2), the latter of which have elemental stoichiometries consistent with alicyclic compounds such as the carboxylic-rich alicyclic material (CRAM) that is posited to make up a significant fraction of oceanic DOM.^65^ These elemental formulas are similar to those photoproduced from light crude oils^66−68^ and from natural riverine and marine DOM.^34,47^ For PE, targeted analyses (e.g., GC-MS) have identified over 200 products of abiotic degradation (i.e., thermal and/or photodegradation; note: studies report no product formation in the dark at room temperature), including alkanes, alkenes, ketones, aldehydes, alcohols, carboxylic acids, keto-acids, dicarboxylic acids, lactones, and esters.^17^ Hydrocarbons (i.e., alkanes and alkenes) and ketones have not been observed as products in water, potentially due to their low solubility.^17^ Hydrocarbons are not detected in our study (i.e., all compounds included oxygen; Figure 2), likely also due to their low solubility, ionization efficiency, and the analytical window we employed to identify photoproducts. Carboxylic and dicarboxylic acids do not absorb light at wavelengths above approximately 300 nm, making them unlikely to photodegrade further in sunlight. They also have low rates of auto-oxidation, while aldehydes, ketones, and alcohols can oxidize to carboxylic acids. Thus, carboxylic and dicarboxylic acids are among the most abundant and stable products of PE photodegradation in sterile water and air^17^ consistent with the high abundance of oxygen-containing aliphatic formulas we observed in DOM generated from PE and PP photodissolution (Table 2; Figure 2).

The assignment of the structure to elemental formulas is imprecise, as the atoms in an elemental formula can be arranged into many different structural isomers. This isomeric freedom allows for acyclic, alicyclic, and aromatic structures to be constructed from the elemental formulas in the “unclassified” category.^34^ Cross-linking and peroxide-radical-initiated reactions can generate alicyclic (e.g., lactones) and aromatic (e.g., phthalates) compounds during PE photodegradation.^17^ Thus, the unclassified formulas identified as photoproducts of PE and PP in our FT-ICR MS data could include acyclic compounds with significant numbers of C–C double or triple bonds, alicyclic compounds, and aromatic compounds.

Photodegradation of the aromatic polymer, EPS, produced the most stoichiometrically, and therefore structurally diverse DOM as indicated by the breadth of compound classes assigned and van Krevelen space occupied by these formulas (Table 2; Figure 2). In addition to the reactions involved in PE and PP photodegradation, ring cleavage reactions can also occur and increase the H/C of EPS photoproducts.^64^ In combination, these diverse reactions are presumably responsible for the widespread of H/C values of DOM generated from EPS (Figure 2). The breadth of H/C values observed for DOM produced from EPS is similar to those reported for photoproducts of DOM from heavy crude oils and residual oil.^67,69^ Ring opening could generate photoproducts with higher H/C than EPS (i.e., formulas with H/C > 1), while cross-linking could generate photoproducts with H/C values below 1 and AI values >0.5, indicating formulas that are likely more aromatic or condensed than the starting polymer (Table 2; Figure 2).

### 3.4 Comparison to Natural DOM and Natural DOM Photoproducts

Photodegradation of natural DOM usually results in the production of aliphatic formulas and unclassified formulas, and the loss of aromatic and condensed aromatic molecular formulas as light-absorbing aromatic moieties are usually the most photolabile.^34,47^ For plastics, photoirradiation of PP and PE produced DOM enriched in aliphatic formulas and unclassified formulas (Table 2; Figure 2). Thus, the photochemical degradation of either natural organics, PE or PP, at the ocean’s surface likely results in chemically similar photoproducts. Photodegradation of EPS yielded not only aliphatic formulas and unclassified formulas but also aromatic and condensed aromatic formulas (Table 2; Figure 2).

### 3.5 Future Directions

The elemental stoichiometry and diversity of dissolved organics generated as PE, PP, and EPS photodegrade in seawater are presented. The use of complementary analytical approaches is required to assess the full diversity of soluble and insoluble organics created from plastic photodegradation. Identifying the chemicals released from plastics as they photodissolve is critical to understanding the fate and impact of plastics and their degradation products in natural waters. This information is required to mitigate the potential negative effects of plastics on both the ecosystem and human health.

## Data Availability

All data needed to evaluate the conclusions in the paper are present in the paper and the Supporting Information.

## Supporting Information Available

van Krevelen diagrams displaying the dissolved organic molecular formulas in the seawater into which plastics were added, and the molecular formulas produced during the photodissolution of the polymers in seawater (Figure S1) (PDF)
Molecular formulas and their abundances for all samples and controls in the study (Table S1) (XLSX)
