# Supplementary material for: Molecular Signatures of Dissolved Organic Matter Generated from the Photodissolution of Microplastics in Sunlit Seawater
Source: Environ Sci Technol. 2023 Nov 13;57(48):20097–106. doi: 10.1021/acs.est.1c03592 (PMC10702502; doi:10.1021/acs.est.1c03592)
Supplement: Supplementary file 1 — es1c03592_si_001.pdf [file es1c03592_si_001.pdf]

Cover Sheet:

Molecular signatures of dissolved organic matter generated from the photo-dissolution of microplastics in sunlit seawater

Aron Stubbins, Lixin Zhu, Shiye Zhao, Robert G. M. Spencer, David C. Podgorski.

Corresponding Author Contact:

Aron Stubbins

Affiliation:

Northeastern University departments of Marine and Environmental Science, Chemistry and Chemical Biology, and Civil and Environmental Engineering

Mailing Address: Hurtig 102, Northeastern University, 360 Huntington Ave, Boston, MA 02115

Email address: aron.stubbins@northeastern.edu. **Telephone:** +1 617-373-5872

Email and mailing addresses for other authors:

Lixin Zhu: lix.zhu@northeastern.edu
Department of Marine and Environmental Sciences, Northeastern University, Boston, MA 02115, USA.
Shiye Zhao: szhao@jamstec.go.jp
Japan Agency for Marine-Earth Science and Technology (JAMSTEC), 2-15 Natsushima, Yokosuka, Kanagawa 237-0061, Japan
Robert G.M. Spencer: rgspencer@fsu.edu
Department of Earth, Ocean and Atmospheric Science, Florida State University, Tallahassee, FL, 32306, USA
David C. Podgorski: dcpodgor@uno.edu
Pontchartrain Institute for Environmental Sciences, Department of Chemistry, Chemical Analysis & Mass Spectrometry Facility, University of New Orleans, New Orleans, LA, 70148, USA

Number of pages in manuscript: 30

Number of Figures in manuscript: 2

Number of Tables in manuscript: 2

SI: One pdf file (contains a single figure, Figure S1) and one Excel file (Table S1. Note the formatting of the Excel file is not preserved well in pdf format, so please use the Excel file).

Figure S1: van Krevelen diagrams displaying the dissolved organic molecular formulas in the seawater into which plastics were added; and the molecular formulas produced during the photo-dissolution of the polymers in seawater.

Table S1: Molecular formulas and their abundances for all samples and controls in the study.

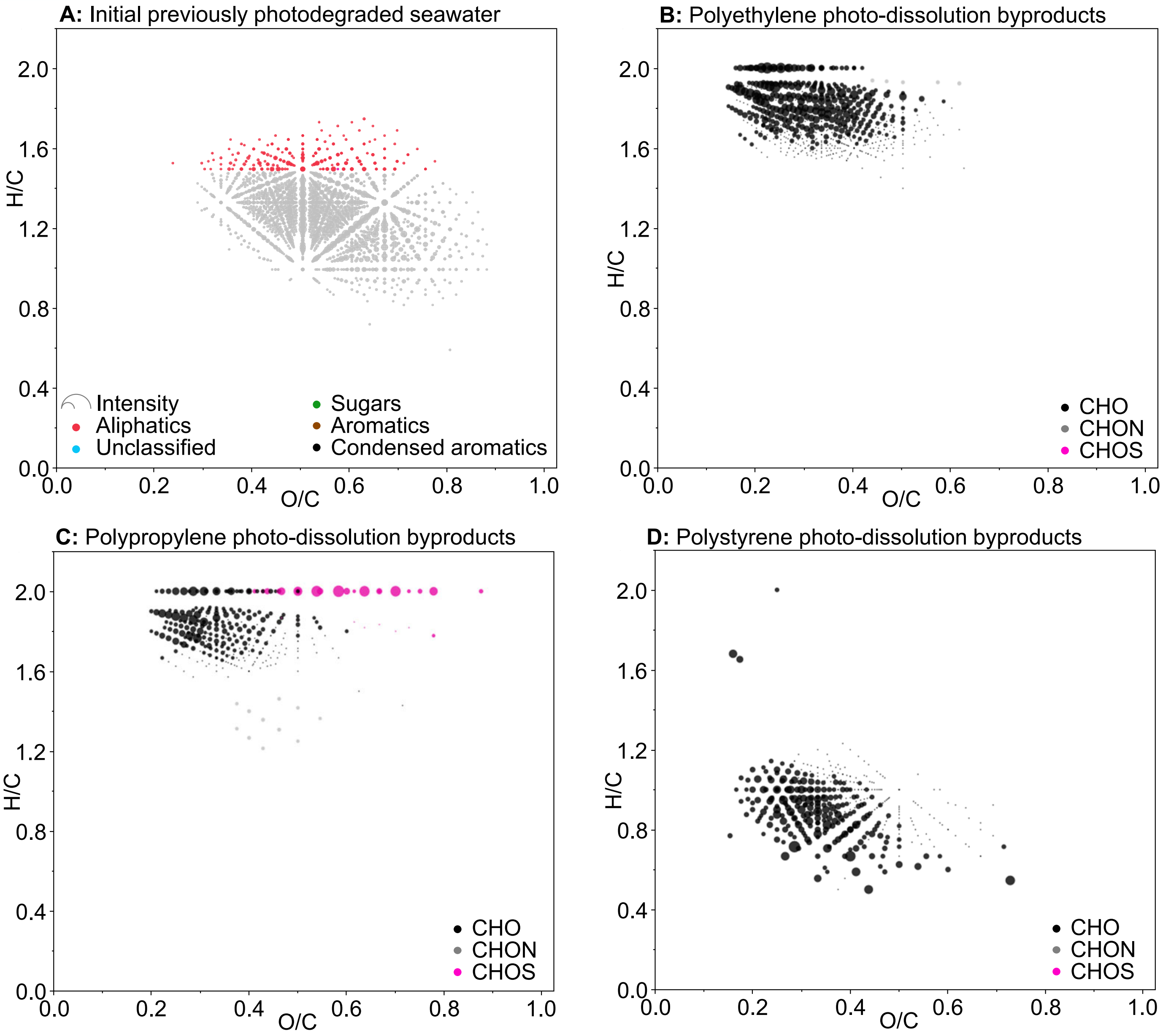

Figure S1: van Krevelen diagrams for **A)** molecular formulas in the previously photobleached and sterile filtered seawater into which plastics were added (Color indicates the compound classes assigned to formulas and the size of the markers indicates the relative abundance of the formula. i.e., a bigger marker indicates a higher intensity peak to which that formula was assigned); and for molecular formulas produced during the photo-dissolution of the following polymers in seawater: **B)** polyethylene; **C)** polypropylene; and **D)** polystyrene, where color indicates the elements present within the formulas.
